# Optimization of Drug Permeation from 8% Ciclopirox Cyclodextrin/Poloxamer-Soluble Polypseudorotaxane-Based Nail Lacquers

**DOI:** 10.3390/pharmaceutics12030231

**Published:** 2020-03-05

**Authors:** Elena Cutrín-Gómez, Andrea Conde-Penedo, Soledad Anguiano-Igea, José Luis Gómez-Amoza, Francisco J. Otero-Espinar

**Affiliations:** Department of Pharmacology, Pharmacy and Pharmaceutical Technology, University of Santiago de Compostela, Santiago de Compostela 15782, Spain; cutgomel@gmail.com (E.C.-G.); andrea.conde.penedo@rai.usc.es (A.C.-P.); s.anguiano.igea@gmail.com (S.A.-I.); joseluis.gomez.amoza@usc.es (J.L.G.-A.)

**Keywords:** transungual drug delivery, nail, medicated nail lacquers, onychomycosis, polypseudorotaxanes, methyl-β-cyclodextrin, poloxamers, ciclopirox olamine, ciclopirox base

## Abstract

Cyclodextrin/poloxamer-soluble polypseudorotaxane-based nail lacquers have demonstrated significant capacity for promoting the permeation of drugs into the nail plate. Furthermore, previous studies have shown that the use of hydroalcoholic blends as vehicles promotes drug permeation. The work described herein studies the effect of the type of alcohol used in the lacquer preparation, and the composition of the vehicle is optimized to obtain soluble doses of 8% and to promote the diffusion of ciclopirox base and olamine across the nail. Permeation studies on different types of alcohols show that optimum results are achieved with short-chain alcohols, and that results become less satisfactory the higher the number of alcohol carbons. In addition, solubility and penetration studies on the bovine hoof have enabled the composition of the lacquer to be optimized for both forms of ciclopirox. The results suggest that optimized lacquers have better ciclopirox diffusion and penetration properties than the commercial reference lacquer. Lastly, in vivo studies in which optimized ciclopirox olamine lacquer was applied for 45 days to the nails of healthy volunteers showed that it caused no negative effects or changes to the nail surface. These results demonstrate the significant potential of cyclodextrin/poloxamer-soluble polypseudorotaxane-based nail lacquers for the ungual administration of drugs.

## 1. Introduction

Onychomycosis is a fungal infection of the nail caused by dermatophytes, nondermatophyte molds, and yeast that can affect 2–18.5%, or higher, of the world’s population [1]. Indeed, in 2003 the Achilles project has shown a prevalence of 26.9% of onychomycosis in Europe [2]. Depending of the infecting fungus specie, the extent and the nail area involvement, and the clinical signs, onychomycosis can present four main clinical types: superficial, proximal subungual, distal lateral subungual, and total onychodystrophic onychomycosis [1,2,3].

Most of today’s topical treatments for onychomycosis are based on nail lacquer compositions containing ciclopirox, amorolfine, or tioconazole [4], and recently efinaconazole and tavaborole has been proposed [4,5,6]. By way of example, in Spain different lacquers with an 8% ciclopirox content are commercially available, amongst which we can cite ciclochem 8% (Ferrer), ciclopirox isdin (Isdin), serra 8% (Serra Pamies), miclast 8% (Pierre Fabre), and onytec 8% (Almirall).

Independently of the drug used, the composition of most of the lacquers sold is based on the use of hydrophobic polymers dispersed or dissolved in volatile organic solvents, designed to promote the formation of even, impermeable films on the nail surface. Examples are Penlac^®^ nail lacquer (Aventis Pharma, Frankfurt am Main, Germany) and Ciclochem 8%, which contain the drug (80 mg ciclopirox; 8%) dissolved in a blend of ethyl acetate and isopropyl alcohol and butyl monoester of poly(methylvinyl ether/maleic acid) as the film-forming polymer. The use of organic solvents is associated with problems as regards their safety, toxicity, complicated disposal and the formation of hydrophobic and impermeable films on the nail surface. The formation of a hydrophobic polymer film in which the active ingredient is dispersed in the form of small, solid precipitates does not favor the delivery of the active ingredient to the nail matrix, thus limiting its penetration. Furthermore, the formation of an impermeable hydrophobic film prevents nail transpiration, which encourages fungal growth. In addition, these types of lacquer require removal between applications using organic solvents or nail files, which causes damage and irritation to the nail and surrounding skin area [7].

Some different physical and chemical enhancement techniques have been proposed to improve the efficacy of topical treatments for onychomycosis. These techniques include the abrasion or the ablation of the nail plate [3], the use of laser treatment [3], iontophoresis [8,9], aqueous or water-alcoholic solutions [7,10,11,12,13,14,15,16], cyclodextrins [15,16], or chemical penetration enhancers [10,17].

Numerous studies suggest that nail hydration plays a highly significant part in the ability of active ingredients to permeate the nail plate [7,10,11,12,13,14,15,16,17]. This has meant that organic lacquers are starting to be replaced by water-soluble bases, which moreover allow better drug penetration into the nail. Furthermore, the new hydrophilic polymers being used have advantages over hydrophobic ones: because they are water-soluble, they hydrate the nail and give a smooth, flexible and matt finish to which patients accept better.

In 2010, Monti et al. [7] compared the bovine hoof diffusion of ciclopirox and amorolfin in a hydroxypropyl chitosan lacquer vehicle with another water-insoluble lacquer (Loceryl^®^, Galderma, Lausane, Switzerland), the composition of which includes a methacrylate acid copolymer, triacetin, butyl acetate, ethyl acetate, and ethyl alcohol. The experimental amorolfin lacquer displayed better diffusion than the commercial water-insoluble polymer option, which suggests better efficacy of the hydroxypropyl chitosan lacquer. Moreover, lacquers prepared with ciclopirox showed better antimycotic efficacy than lacquers prepared with amorolfin. 

In 2013, Nogueiras Nieto et al. [11] developed an aqueous lacquer for the treatment of onychomycosis and nail psoriasis, the composition of which includes soluble polypseudorotaxanes of Pluronic F127^®^ and β-cyclodextrin derivatives, with the aim of improving the penetration of antimycotic agents and corticoids into the nail. The presence of the poloxamer facilitates the formation of thermosensitive hydrogels in situ on the nail surface, and their combination with soluble cyclodextrins increases the solubility of the drugs contained in the lacquers. In the study described herein, the authors propose the inclusion of acetylcysteine to enhance nail absorption, since it favors nail hydration and increased porosity, in turn favoring permeation of the drug [10]. The drawback of using acetylcysteine is that it is a labile molecule with poor chemical stability, which releases an unpleasant odor characteristic of products with sulfhydryl groups. Another drawback of these lacquers is that, in spite of containing poloxamers and cyclodextrins, their solubilization capacity remains limited, and the 8% concentration normally used in commercially available ciclopirox lacquers is not attained. In an attempt to improve lacquer solubilization and penetration, in 2018 Cutrín-Gómez et al. [16] studied the effect of different soluble cyclodextrins at different concentrations. They found that both partially methylated and hydroxy-propylated derivatives of β-cyclodextrin were able to interact with nail components, promoting hydration of the nail and the formation of a porous structure that favors permeation of active ingredients into the nail plate. Furthermore, higher cyclodextrin concentration increases the soluble dose in the lacquer. However, no differences were observed between lacquers prepared with both varieties of cyclodextrin or between the 10% and 20% concentrations in bovine hoof ciclopirox permeability. 

To dissolve better still the lipophilic active ingredients, in 2018 Cutrín-Gómez et al. [15] studied the effect of including alcohol on drug penetration of the nail using soluble polypseudorotaxane lacquers. The authors were surprised to find that when the water component was replaced with water–ethanol mixtures, instead of reducing penetration, which could be expected since ethanol should reduce hydration, what actually happened was that the rate and degree of penetration of ciclopirox olamine and clobetasol propionate were significantly increased in the bovine hoof model and human nail. However, in the study described herein with the proportions of ethanol studied, ciclopirox concentrations of around 2% were attained in the lacquers, far short of the 8% concentration found in commercially available lacquers.

This study, based on the latter formulations, examines how the type of alcohol used in the formulation affects drug permeability, the optimization of vehicle composition to achieve a concentration of 8% ciclopirox base in the lacquers, and examines its effect on drug diffusion and nail penetration, using as reference formulation a commercially available hydroalcoholic lacquer containing chitosan in its base and 8% ciclopirox (Onytec ^®^, Alfasigma, Alanno, Italy). In addition, an in vivo study was carried out on healthy volunteers to determine any changes in the nail structure as a result of prolonged treatment with optimized lacquers. 

Ciclopirox is a synthetic antifungal drug (CAS number 29342-05-0) with a molecular weight of 207.27 g/mol, logP 2.59, pKa 6.84, and aqueous solubility of 0.22 mg/mL. Ciclopirox olamine is a salt of ciclopirox with a molecular weight of 268.35, logP 2.15, and aqueous solubility of 14.41 mg/mL [18].

## 2. Materials and Methods 

### 2.1. Materials

Pluronic^®^ F127 (Sigma-Aldrich, St. Louis, MI, USA), hydroxypropyl-β-cyclodextrin (molar substitution 0.65 and molecular weight 1399 Da, Kleptose HPB^®^, Roquette, Lestrem, France), sodium lauryl sulfate (Fagron Iberica, Terrasa, Spain), ethanol (Merck Millipore, Burlington, MA, USA), distilled water (Elix^®^ Merck Millipore, Madrid, Spain), ciclopirox (PCAS, Turku, Finland), and ciclopirox olamine (Erregierre S.p.A, Sant Paolo D’Argon, Italy) were used. Bovine hooves were obtained from the local abattoir (Compostelana de Carnes S.L; Santiago de Compostela, Spain). They were thoroughly cleaned with water and hydrated for 24 h to facilitate obtaining fine slices (0.3–0.7 mm thick) (Ufesa Professional Slicer FS50). For the study, we selected slices of even thickness free from defects or splits.

The saline phosphate buffer solution was prepared as per the 8th Edition of the Pharmacopoeia European using potassium phosphate dihydrogen, sodium chloride, and sodium dihydrogen phosphate dodecahydrate, all of analytical grade. The sodium azide added to the buffer solution to prevent microbial growth was supplied by Panreac Quimica SA (Barcelona, Spain), and methanol (Prolabo) was used to extract the ciclopirox olamine that penetrated the hooves and nails. 

Ethanol, butanol, hexanol, and octanol were from Sigma-Aldrich, Germany.

### 2.2. Methods

#### 2.2.1. Preparation of Lacquers 

Lacquers were prepared with 5% Pluronic^®^ F127, 10% HPB, 1% sodium lauryl sulfate, and different water and ethanol ratios, and the addition of ethyl acetate was tested in order to dissolve 8% ciclopirox in base or salt form, obtaining stable lacquers without any nucleation or precipitation taking place.

To prepare the lacquers, first the HPB was dissolved in water or a water–ethanol mix under constant agitation. The solution was then cooled to around 4 °C to promote the correct dissolving and homogenization of the poloxamer (Pluronic^®^ F127). Under constant agitation, ethyl acetate and sodium lauryl sulfate were added. Lastly, ciclopirox olamine or ciclopirox base 8% was added. Ciclopirox concentrations in the lacquers were determined by UV spectrophotometry (Hewlett-Packard 8452A, Palo Alto, CA, USA).

#### 2.2.2. Study on the Effect of Type of Alcohol on Nail Diffusion of Ciclopirox

Lacquers of identical compositions to that described in the section above were prepared, but with a water-to-alcohol ratio of 1:1. In this case, the amount of ciclopirox incorporated was lower (2%) due to it being impossible to fully dissolve the 8% dose. 

Ethanol, butane, hexanol, and octanol were used in this study, and the amount of ciclopirox base diffusing into in the bovine hoof model was measured over 6 days. 

#### 2.2.3. Permeability Studies and Measurement of Ciclopirox in Hoof and Nail

In view of the difficulty of obtaining human nail samples of sufficient size for carrying out diffusion studies, in the first stage of evaluating the different lacquer vehicles, bovine hoof models were used. 

The samples were hydrated for two hours, then dried and arranged between two cylindrical polytetrafluoroethylene (PTFE) adaptors (Mecanizados del Noroeste, Santiago de Compostela, Spain), giving an effective diffusion area of 0.196 cm^2^. This arrangement was assembled in Franz vertical diffusion cells (Vidrafoc, Barcelona, Spain) with a receptor compartment volume of 7 mL. The dorsal and ventral surfaces of the nails were arranged facing the donor and receptor compartments, respectively. The donor compartment contained 2 mL lacquer. The receptor medium was a pH 7.4 saline phosphate buffer. Sodium azide (30 mg/l) was added to prevent microbial and algae growth. The receptor compartment was kept at a constant temperature (37 ± 0.5 °C). At 24 h intervals, 1 mL samples were taken from the receptor compartment, this volume being replaced with fresh receptor medium.

The concentration in the receptor medium was quantitated following filtration through nylon filters. Ciclopirox was determined by UV spectrophotometry (Hewlett Packard 8452A diode array spectrophotometer) at 308 nm.

The thickness of each hoof was measured using a micrometer (Mitutoyo) before the experiments began. The cumulative amounts diffused into the receptor compartment were standardized for an area of 0.049 cm^2^ and plotted against time.

At the end of the diffusion studies, the amount of ciclopirox present in the hooves was measured. To do so, the diffusion cells were dismantled, and the samples carefully cleaned with distilled water and dried with cellulose paper. Then, the sections of hooves or nails that had been exposed to lacquer were separated and cut into small pieces that were weighed in vials. Then, 5 mL of 5% methanol solution was added to each vial, and they were incubated under agitation for 6 days at 25 °C so that the drug could be extracted. A 1 mL sample was taken from each vial and filtered through 0.45 µm nylon filters, and the drug was quantitated as described above. 

#### 2.2.4. In Vivo Study on Healthy Volunteers 

The study was carried out on 14 healthy male and female volunteers presenting no nail damage or alteration and without relevant derma conditions. The study was carried out as per the criteria laid out in the Structure and Content of Clinical Study Reports from ICH Harmonized Tripartite Guideline, International Recommendations IH Topic E6, CPMC/ICH/135/95 of May 1st 1996, European Parliament and Council Guideline 2001/20/EC-DOCE of May 1st 2001. Each volunteer was informed beforehand about the type and procedure of the study and signed an informed consent form.

The optimized nail lacquer containing ciclopirox olamine was applied once daily to the nails on one hand of the volunteers for a period of 45 days. After this period had elapsed, pieces of the treated nails were cut (2–3 mm size), and pieces of the untreated nails on the other hand were taken for control purposes. Samples were fixed to microscope slides with an adhesive. An S neox non-contact 3D Surface Profiler from SENSOFAR Surface Metrology (Barcelona, Spain) was used in confocal mode to acquire 3D images using the Nikon EPI 20X v35 lens and with the following parameters: topography, 1360 × 1024 p; area, 877.20 × 660.48 μm; ZSCAN, 100–200 μm; acquisition time, 00:30–01:30 min.; pixel size, 0.64 μm/pixel.

Surface roughness was characterized using the 3D (S-parameters) and functional (volume) parameters according to ISO 25178 (Table 1): 

## 3. Results and Discussion

The bovine hoof model has been used in different works in permeation studies as an alternative to human nail. Microstructural characterization of bovine hoof and human nail has shown that the bovine hoof surface is more porous than the nail surface, although the internal porosity is similar for both substrates [10]. Nevertheless, the hydration modifies the porous microstructure of nails and hooves, increasing the pore size and promoting the pore interconnection. This effect is more pronounced in hooves probably because hoof proteins have fewer cystine residues and disulfide links than human nail [19,20]. Due to these differences, bovine hooves have been reported to be more permeable than human nails, and so caution is recommended when data obtained with this animal model are extrapolated to human nail [10]. Our previous results showed that onychomycotic nails have higher porosity and lower amounts of disulfide bonds compared to healthy nails [21], and that the presence of dermatophyte fungi makes the nail plate more permeable to water and drugs similarly to bovine hooves. In this sense, Monti et al. in 2011 validated the bovine hoof as a model for infected human toenail to study in vitro ciclopirox transungual permeation [22]. Additionally, there are different previous works that demonstrate the usefulness of the bovine hoof model to establish comparisons in the ability to penetrate the nail of drugs from different formulations [11,15,16,17].

To conduct diffusion experiments, we used two cylindrical PTFE adapters with an O-shaped ring of 0.5 cm diameter, giving an effective diffusion area of 0.196 cm^2^, and the bovine hoof slices used were 0.8 cm in diameter. Palliyil et al. in 2014 [23] demonstrated the importance of the effect of the passive lateral diffusion of ciclopirox olamine in nail during in vitro permeation experiments. This work shows lateral diffusion can reduce the in vitro passive transungual drug permeation, and they highlight the importance of the relationship between the dimensions of the nail sample and the diffusional surface, recommending a nail just enough to cover the hole of the adapter. In our case, the ratio between the area of the hole of the adapter to the area of the bovine hoof slice was 0.392, higher than the ratio 0.283 used for Palliyil et al. with the smallest nail samples. Therefore, a great influence of lateral diffusion on our results is not expected.

**Figure 1:**
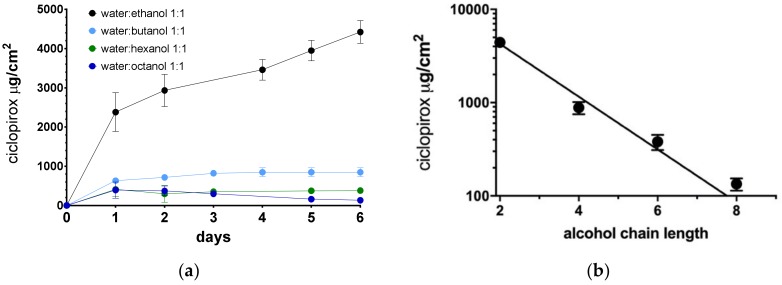
Diffusion kinetics (**a**) and the relationship between amount of ciclopirox diffused across hoof membrane after 6 days (**b**) and length of alcohol chain used in lacquer preparations.

Figure 1 shows the diffusion kinetics of ciclopirox base in the hoof membrane model and the relationship between the total amount of ciclopirox base diffused at day 6 obtained with different types of alcohol used in the lacquer preparations. We found a linear logarithmic relationship between the diffused drug and the length of the alcohol chain. It can be seen that a longer alcohol chain length significantly reduced the amount diffused. 

Walter et al. [24,25] studying the influence of the length of carbon chain in the nail diffusion of different alcohols observed an exponential decrease in the permeability and diffusion coefficients of these alcohols through the nail with increasing chain length. The authors explained the decrease in the diffusion rate by the increase of the resistance of the keratin matrix to diffusion of more lipophilic, longer chain length alcohols, and by the reduced hydration of nails due to the effects of these alcohols. In our case, we observed also an exponential decrease in the amount of ciclopirox diffused across the hoof membrane with the length of the alcohol carbon chain used in the preparation of the vehicle. This decrease may be caused by a reduction in the hydration and swelling of the hoof structure, producing a reduction in the hoof matrix porosity. Previous work has shown the importance of the porosity and pore distribution of the nail matrix in the nail permeability of antifungals and corticosteroids [10,15,16,17]. Therefore, of all the alcohols tested, ethanol was the most suitable for inclusion in lacquer preparations. 

**Figure 2:**
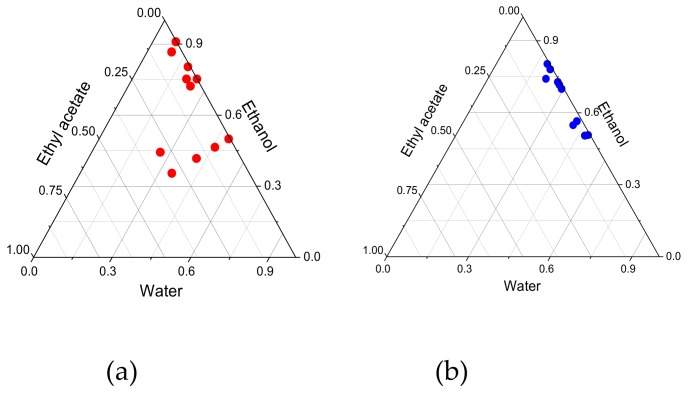
Ternary diagrams showing the proportions in which 80 mg/mL ciclopirox olamine (**a**) and base (**b**) are dissolved.

Once ethanol was selected, the next stage in lacquer optimization was to determine the water and ethanol ratio necessary for ensuring that the 8% ciclopirox dose dissolves, and to promote high permeability. A third volatile solvent, ethyl acetate, was included in the vehicle in order to obtain bases that spread easily across the nail and evaporated relatively quickly for enhanced patient acceptance. Based on a composition of 10% HPB, 5% Pluronic^®^ F125, and 1% SLS, different vehicles were prepared to change the proportions of water, ethanol, and ethyl acetate in the blends. For the purpose of their evaluation, direct macroscopic observation was performed to check in which blends the 80 μg/mL dose of ciclopirox olamine or ciclopirox base was fully dissolved, subsequently measuring in same the final concentration by means of UV–vis spectroscopy to confirm that it had fully dissolved. Ternary diagrams were drawn using the results in order to establish the areas of interest (Figure 2). 

As can be seen, the ciclopirox olamine dose dissolved adequately even at a high water concentration, and ethyl acetate was not necessary for ensuring that it does so correctly. By contrast, ciclopirox base required ethanol concentrations of over 48% for good results to be obtained. Taking these results into account, different lacquers were prepared with compositions featuring the areas of interest in the ternary diagrams, and the ungual permeability of ciclopirox was assessed using the bovine hoof model.

**Figure 3:**
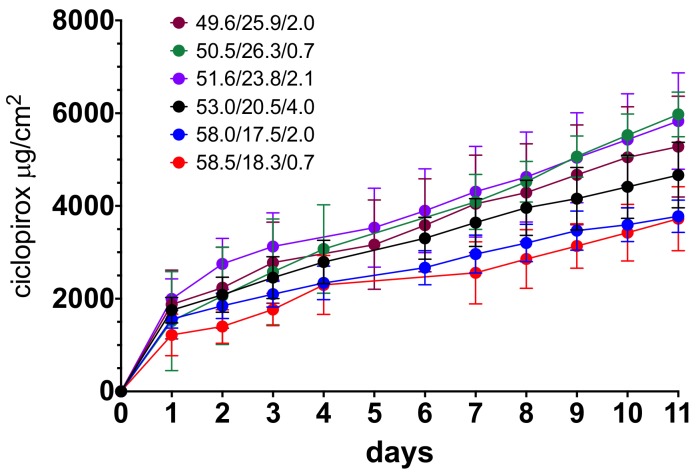
Diffusion kinetics of ciclopirox base through bovine hoof. Symbols indicate the ethanol/water/ethyl acetate proportion in the blends.

Figure 3 shows the base hoof permeation kinetics of ciclopirox over 11 days for different vehicle compositions. The penetration rate was highest during the first day of lacquer application, and the flux stabilized thereafter, showing a linear increase over time of the amount diffused. 

**Figure 4:**
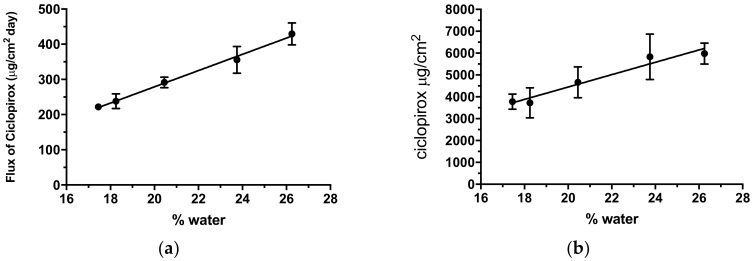
Relationship between ciclopirox flux from day 1 to day 11 (**a**) and the total amount of ciclopirox base diffused by the end of day 11 (**b**) according to the proportion of water in the vehicle.

Nail permeation kinetics showed that a higher proportion of water in the blend led to a higher amount of drug being diffused. To investigate the relationship between the amount of water and trans-hoof diffusion, Figure 4 shows the amount of ciclopirox standardized by the diffusion area diffused after 11 days and the ciclopirox flux, obtained via linear regression fitting of the amount diffused between the first day and day 11, compared with the proportion of water used in vehicle preparation. A linear increase in flux (R^2^ = 0.9952) and amount diffused (R^2^ = 0.9520) can be observed as the proportion of water in the blend increased. A previous study [15] showed that, surprisingly, the substitution of water by hydroalcoholic blends in a cyclodextrin/poloxamer-soluble polypseudorotaxane-based vehicle improved the hoof and nail penetration of ciclopirox olamine and clobetasol propionate. In this study, water-to-ethanol ratios of 1:1; 1:2; 1:3, and 1:4 were tested, and it was found that increasing the proportion of ethanol to above 50% did not improve lacquer permeability. In our case, the proportion of ethanol had to be increased in order to fully dissolve the 80 mg/mL ciclopirox required for an 8% dose. Figure 4 shows that ciclopirox diffusion tends to increase as the proportion of water in the blend increases, but no effect was observed regarding the amount of ethyl acetate in the blend for the proportions tested. 

The increase in diffusion observed when the proportion of water ions the vehicle increased may be associated with nail hydration capacity and increased chemical activity of the drug, promoting its trans-hoof diffusion. Lack of ethyl acetate on diffusion may be associated with the solvent’s rapid evaporation once the dose was administered in the donor chamber of the diffusion cells. This may be advantageous because it would allow the lacquer to dry more quickly after application to nails and would have no effect on drug penetration.

**Figure 5:**
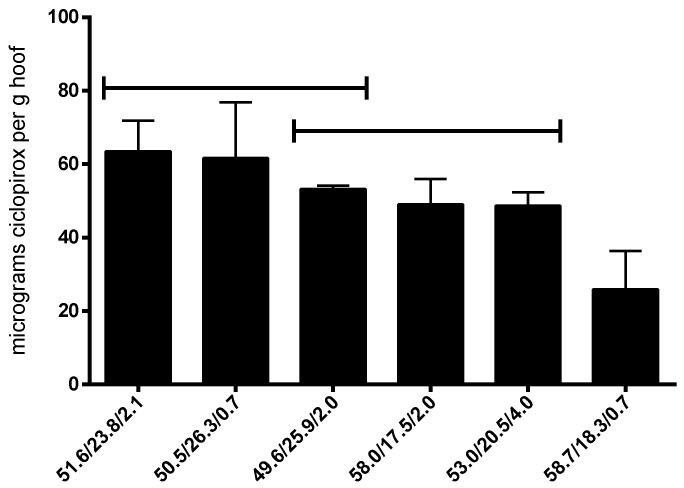
Amount of ciclopirox base found in the hoof after 11 days of the diffusion experiment. Black lines represent homogeneous groups (one-way ANOVA and Tukey test, α < 0.01).

Figure 5 shows the amount of ciclopirox in the hoof after 11 days of the experiment. The graph shows a tendency to decrease the amount of drug accumulated in the bovine hoof from lacquers made with lower amounts of water (<25%).

**Figure 6:**
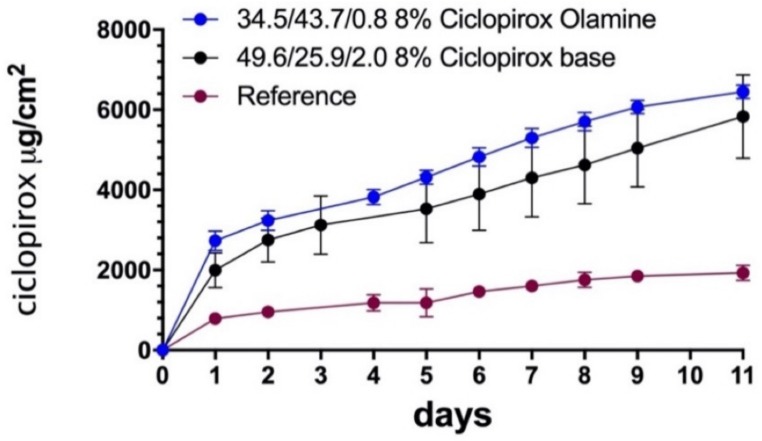
Trans-hoof diffusion kinetics of ciclopirox olamine and ciclopirox base, compared with the same for the commercial reference lacquer used.

**Figure 7:**
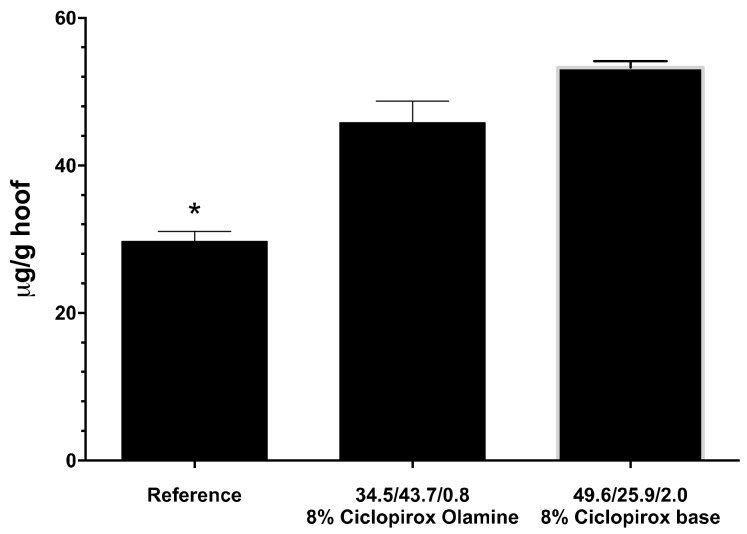
Amount of ciclopirox base found in hooves after 11 days of the diffusion experiment. *Significant differences between means for an α < 0.05.

As Figure 2 shows, in the case of ciclopirox olamine, the proportion of water may be increased without compromising drug solubility in the lacquer. Takings these results into account, a lacquer was prepared to contain 8% ciclopirox olamine, 34.5% ethanol, 43.7% water, and 0.7% ethyl acetate, and a drug diffusion study was carried out on the bovine hoof model. Figure 6 and Figure 7 show the diffusion profiles and amount of drug penetrating the hoof. Two-way variance analysis of the diffused amount in hoof in the time, and subsequent Tukey multiple comparison tests, showed that there were no significant differences between diffusion of ciclopirox base and ciclopirox olamine from optimized bases, and that both provide significantly higher diffusion values than the commercial reference lacquer for all times tested (α < 0.05). Similarly, the concentration of ciclopirox found in the hoof after 11 days of the experiment was significantly higher for lacquers prepared with cyclodextrin/poloxamer-soluble polypseudorotaxane than for those prepared with hydroxypropyl-chitosan, regardless of whether the drug was in its base form or in ciclopirox olamine form (one-way ANOVA, Tukey test, α < 0.05). Baswan et al. in 2016 [26], studying the passive diffusion of 42 unchanged solutes in hydrated human nail plate, observed that there is an inverse dependence of nail permeability with molecular weight but not apparent dependence with logP. Ciclopirox base and ciclopirox olamine have comparable molecular weights and slight differences in logP values (2.59 vs 2.15). This is in concordance with the similar values of permeability observed in the bovine hoof from the hydroalcoholic lacquers. The differences observed in the hoof penetration of ciclopirox with the reference lacquer should, therefore, be attributed to the vehicle’s activity. These results confirm the superiority of the optimized lacquers prepared in our study over the commercial lacquer and, therefore, their huge potential for treating onychomycosis. 

The amounts of ciclopirox and ciclopirox olamina recovered from the bovine hoof slices after the experiment were 29.7 ± 0.36, 45.8 ± 2.93, and 53.26 ± 0.89 for reference, 34/43.7/0.8 8% ciclopirox olamine, and 49.6/25.9/2.0 8% ciclopirox base, respectively. According to Hui et al. [27], the fingernail density is 1.33 g/mL. If we assume that it is similar for the bovine hoof, the drug concentrations in the slices are 39.5, 60.9, and 70.8 µg/mL, which are higher than the minimum inhibitory concentrations (MICs) of ciclopirox for *Trichophyton rubrum* DSM 4167 (15.6 µg/mL) [28] or seven *T. rubrum* strains (24.13 µg/mL) [29].

**Figure 8:**
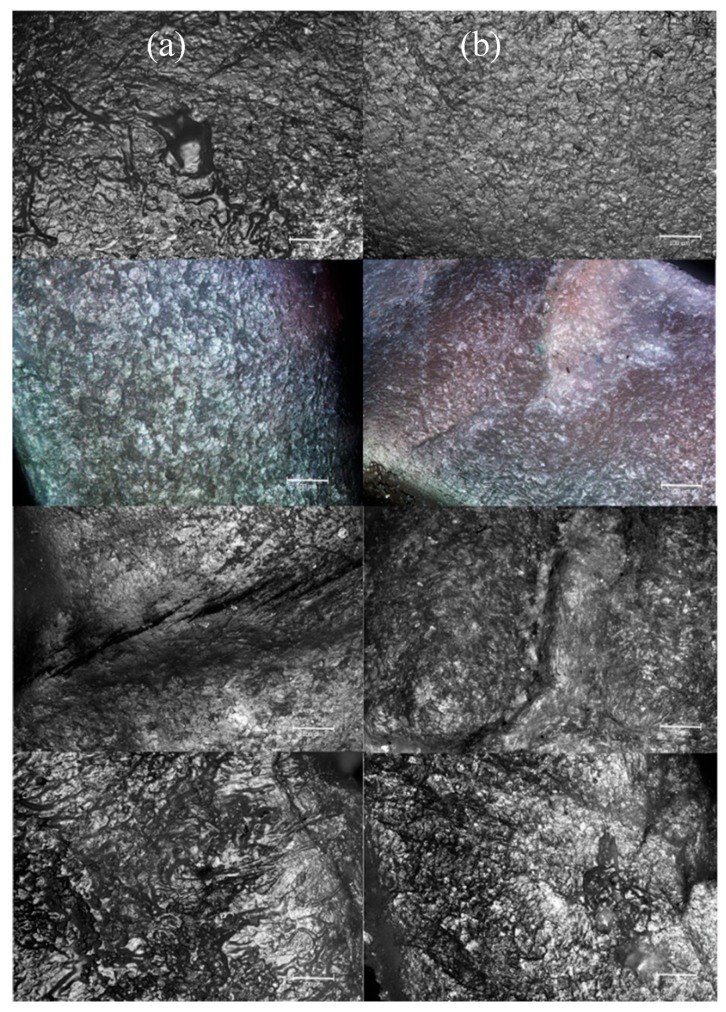
Confocal optical microscopy micrographs of the nail surfaces of some volunteers. (**a**) Treated nails, (**b**) untreated nails.

**Figure 9:**
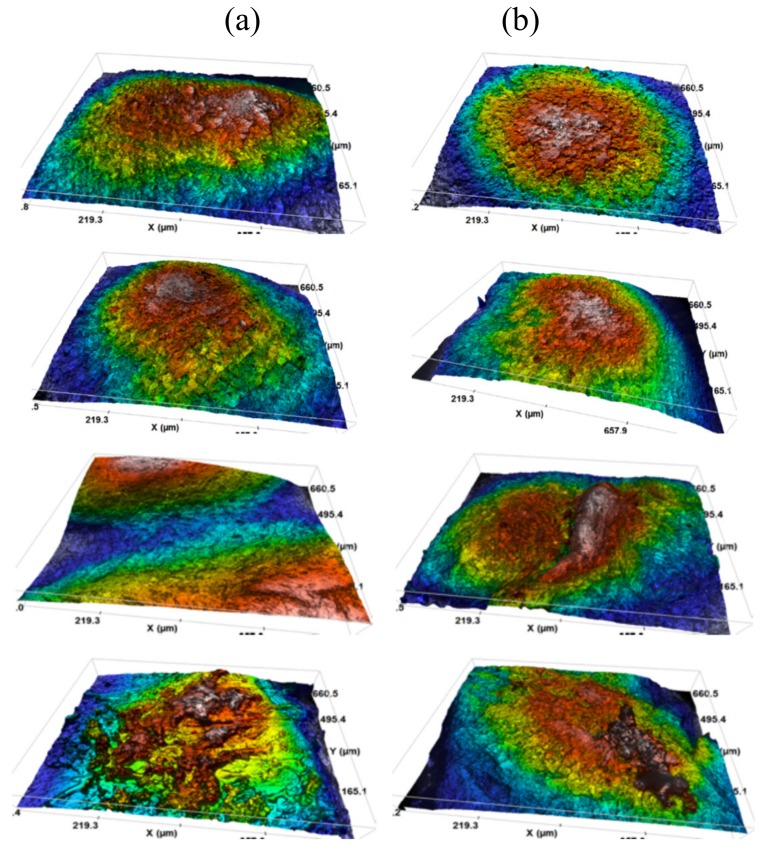
Images of the models of the dorsal surface of the nails of several volunteers obtained by non-contact 3D surface profilometry. (**a**) Treated nails, (**b**) untreated nails.

With a view to studying any changes in nail surface structure because of prolonged treatment with the optimized lacquer, the formulation containing ciclopirox olamine was administered daily to 14 volunteers over a period of 45 days, and their nails were analyzed using non-contact 3D surface metrology based on confocal optical microscopy. Figure 8 and Figure 9 show some confocal images and 3D surface models obtained from treated (Figure 8a) and untreated (Figure 8b) nails of several volunteers. The confocal images of nails treated with the optimized lacquer showed the appearance of a smooth, continuous material on the nail surface, which is the film of polypseudorotaxanes that forms on the nail surface following lacquer application. 

Using the 3D images shown in Figure 9, and after correcting the natural curve of the nails to prevent interference in surface calculations, the values of the main 3D roughness (S-parameters) and functional (volume) parameters were determined (Table 2). Using these values, the relationship between the values obtained for treated and control nails (untreated nails of the same volunteer) was determined. 

**Figure 10:**
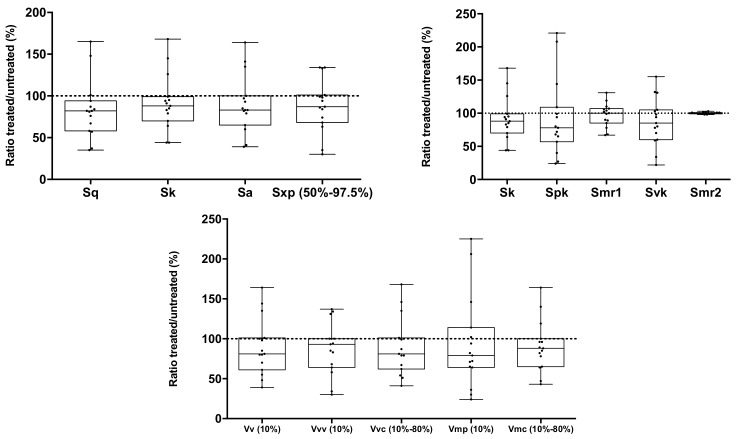
Box-and-whisker charts showing the relationship between the values of 3D parameters (S-parameters) and functional (volume) parameters of treated and untreated nails.

Figure 10 shows these relationships expressed in % in a box-and-whisker chart. We can see that for most of the volunteers, there was a slight lowering of the roughness values studied, and that 75% of the values obtained for treated nails were below the values that would be for similar roughness in untreated control nails. The median values for most of the parameters suggested a slight lessening in nail roughness following continuous administration of the optimized lacquer, probably due to its film-forming effect on nails, which reduces their natural roughness. However, the results of the paired-sample T-test undertaken to compare the values obtained for treated and control nails showed that there were no statistically significant differences in roughness following daily application of the optimized lacquer over a period of 45 days. The study, therefore, confirms that the optimized nail lacquer developed respected the nail structure following continuous and repeated application in prolonged courses of treatment and did not damage or alter the nail surface structure.

## 4. Conclusions

The permeability studies carried out on cyclodextrin/poloxamer-soluble polypseudorotaxane lacquers prepared using different alcohols have demonstrated the superiority of short-chain alcohols in promoting drug permeability in the bovine hoof model. A decreasing linear logarithmic relationship was observed between the amount of ciclopirox diffused per unit of area and the number of carbons in the alcohol carbon chain. In view of the lower topical toxicity and permeability values observed, ethanol appears to be the most appropriate alcohol for preparing these types of vehicles. Taking these results into account, the composition of the lacquer base was optimized to achieve a soluble dose of ciclopirox base and ciclopirox olamine of 8%, and to obtain better nail permeation efficacy. In the case of ciclopirox base, given its lower solubility in the lacquer, higher proportions of ethanol need to be used. However, diffusion results show that a higher proportion of alcohol is detrimental to permeability. Therefore, both solubility and permeability must be taken into consideration when selecting the proportion of alcohol. The results obtained show that by selecting the appropriate composition for the hydroalcoholic base of the nail lacquer, diffusion profiles can be optimized, and significantly higher levels of nail penetration by anti-mycotic agents can be achieved than those obtained with the commercial reference lacquer.

Furthermore, the results of in vivo studies obtained after daily administration of the optimized lacquer to healthy volunteers over prolonged periods show that the lacquer is safe and does not alter the nail surface structure.

These results demonstrate the huge potential of the cyclodextrin/poloxamer-soluble polypseudorotaxane base for ungual administration of antifungal medicines. Nevertheless, more in vitro and in vivo studies are necessary to validate the results obtained using the bovine hoof model.

## 5. Patents

This work is part of the family patent application EP 15725664 A 20150603 “Hydroalcoholic system for nail treatment”.

## Figures and Tables

**Table 1:** 3D (S-parameters) and functional (volume) parameters according to ISO 25178 used to characterize nail surface roughness.

Parameter	Description of Parameter
S_Q_	Root mean square height of surface
S_K_	Roughness depth
S_A_	Arithmetical mean height of surface
S_XP_	Maximum height
S_PK_	Reduced maximum height
S_SK_	Skewness of height distribution surface
S_MR1_	Portion of material from peaks
S_MR2_	Portion of material from valley
S_VK_	Roughness of surface depressions
V_V_	Void volume
V_MP_	Peak material volume
V_MC_	Core material volume
V_VC_	Core void volume
V_VV_	Valley void volume

**Table 2:** 3D (S-parameters) and functional (volume) parameters according to ISO 25178 obtained to characterize the surface roughness of human nails (mean ± SD, n = 14).

Parameter	Treated Nail	Control
S_Q_	1.95 ± 1.05	3.74 ± 5.08
S_K_	4.07 ± 1.84	4.93 ± 2.62
S_A_	1.44 ± 0.66	1.82 ± 1.00
S_XP_	4.26 ± 2.01	5.47 ± 3.07
S_PK_	2.67 ± 1.64	3.58 ± 2.13
S_SK_	2.67 ± 1.64	3.58 ± 2.13
S_MR1_	10.17 ± 1.57	10.60 ± 2.03
S_MR2_	87.80 ± 0.84	87.67 ± 1.17
S_VK_	2.87 ± 1.46	3.87 ± 2.26
V_V_	2.28 ± 1.05	2.95 ± 1.79
V_MP_	0.14 ± 0.08	0.18 ± 0.10
V_MC_	1.47 ± 0.66	1.81 ± 0.99
V_VC_	2.01 ± 0.92	2.60 ± 1.61
V_VV_	0.28 ± 0.13	0.36 ± 0.20
